# Problems faced by patients and health service utilization experiences of gastrointestinal patients during lockdown due to COVID-19 pandemic

**DOI:** 10.12669/pjms.38.3.4799

**Published:** 2022

**Authors:** Hamid Ali Kalwar, Lubna Kamani

**Affiliations:** 1 Hamid Ali Kalwar, MBBS, FCPS (Gasto). Consultant, Indus Hospital, Karachi, Pakistan; 2 Lubna Kamani, MBBS, FCPS, MRCP (UK), FRCP (Lond), FACG. Associate Professor & GI Residency Director, Gastroenterology Department, Liaquat National Hospital, Karachi, Pakistan

**Keywords:** Health service utilization, COVID-19 pandemic, Lockdown, Gastroenterology, Telemedicine

## Abstract

**Objectives:**

The COVID-19 pandemic undermined the health service delivery and utilization of essential health care services globally. The current study therefore aimed to explore the health-seeking behaviors and challenges faced by patients for the management of gastrointestinal diseases.

**Methods:**

A cross-sectional study was conducted at the outpatient department of Gastroenterology, Liaquat National Hospital, Karachi from March 2020 to July 2020 during the COVID-19 lockdown phase to explore patient experiences. Data was collected using a survey questionnaire. All patients of either gender were included after informed consent. Statistical analysis of the data was conducted using SPSS 21.0.

**Results:**

A total of 184 patients were included who visited the hospital to seek medical services during the COVID-19 lockdown phase. The mean age of the population was 42.7 years (±16.13). Of these, n=94 (51.1%) were males All patients had gastrointestinal issues with different comorbid conditions. One forty-seven n=147 (79.9%) presented with active complaints whereas, n=37 (20.1%) patients visited the hospital for their follow-up checkup. Out of 184 patients, n=33 (17.9%) patients reported of having fear of visiting hospital due to COVID-19 outbreak. A statistically significant difference *p<*0.001 was noted between the history of comorbidities and patient delaying a visit to the healthcare due to the fear of COVID-19. Additionally, 61 (73.5%) patients with co-morbidity faced difficulty in finding public transport (p=0.01). Nevertheless, n=171 (93.0%) patients expressed satisfaction with the services provided by the hospital during the lockdown phase.

**Conclusion:**

Patients with gastrointestinal conditions were largely affected by lockdown largely due to fear of contacting COVID-19 disease and inaccessibility to the public transportation. Widely available telemedicine service might overcome these shortcomings and ensure continuity of quality care.

## INTRODUCTION

The COVID-19 pandemic caused widespread disruption in health, social, and economic spheres across the globe with drastic public health policy measures adding to the adversity.^1^ Although some weaknesses in pandemic risk management capacities of all countries were highlighted by global health monitoring bodies and the Global Preparedness Monitoring Board Report of 2019^2^-^4^ no significant efforts were taken by the governments to prepare for an infectious disease outbreak of this scale. Nevertheless, the novelty of the pathogen and its epidemiology has been a major factor resulting in uncertainties and delays ineffective management strategies.^4^ However, considerable heterogeneity in the degree of responsiveness and timing entailed varied public health outcomes across and within regions despite the implementation of overlapping response and management strategies.^5^

The current pandemic response not only dismantled healthcare systems but its impacts on population health are far-reaching ranging from hindrances in essential health services like routine immunization, treatment and management of other acute and chronic conditions, maternal and child health services, mental health services, and collapsing of healthcare systems.^5^-^9^ On the other hand, the negative impact on the socioeconomic domain of the societies resulted in unemployment, reduced individual productivity, business shut-downs, tourism suspension, disruption in routine education systems and supply chains, political issues, disruption of public transport systems, and shortages of human resources for health.^10^–^12^ While the health impacts of the pandemic are inevitable, a very high proportion of the population, particularly those who had existing chronic diseases faced immense hardships in seeking medical services amidst restrictive policies for pandemic management.^1^,^13^ Modelling scientists indicated amplification of limitations and trade-offs in routine healthcare services during the peak of the pandemic as the efforts of policy-makers and healthcare departments were in favour of outbreak management and containment.^14^,^15^

Significant reductions in attendance at healthcare facilities and utilization of reproductive health services, maternal and child healthcare, immunization coverage, and uptake of HIV/AIDS services were recorded during the Ebola Virus outbreak.^16^ These attitudes are further precipitated when there are shortages of personal protective equipment, perceptions of contracting the infectious disease at the routine healthcare facility, a high prevalence of COVID-19 related anxiety and fear, and a lack of trust in the service standards.^17^,^18^ Fear of contacting COVID-19 during routine healthcare visits to the hospitals has been documented as a major limiting factor to preventive as well as curative health service utilization in many countries.^19^

In Pakistan, the health service utilization for acute and chronic conditions also diminished due to fear of exposure with the potential disease carriers and accessibility issues as due to lockdowns and closure of routine transportation services, people faced difficulty in reaching the tertiary-care hospitals.^20^ The current study, therefore, aimed to explore the health-seeking behaviors of the patients visiting a tertiary healthcare facility for the management of acute and chronic conditions. The study also attempted to identify the challenges faced by patients in health services utilization during COVID-19 pandemic.

## METHODS

A cross-sectional study was conducted at the Outpatient Department of Gastroenterology, Liaquat National Hospital, Karachi from March 2020 to July 2020 during the COVID-19 lockdown phase after approval of hospital ethics committee. A total number of 184 patients of either gender were included after informed consent.

### Data Collection

Primary data were collected using a survey questionnaire including details about the issues faced by the patients in utilizing the hospital services during the pandemic-related lockdown. This included questions related to the reason for the hospital visit, active complaint and its duration, co-morbid conditions health-seeking details, availability of local physician, information related to delay in services, waiting in the emergency room (ER), type of emergency treatment received, denial for prescription, use of personal protective equipment/mask by the patients and the attendants, types of personal protective equipment used by the patients and the attendants, fear of visiting the facility due to COVID-19, modes of transportation and issues faced in reaching the hospital, and the patients’ satisfaction with the hospital services.

### Statistical Analysis

SPSS version 21.0^21^ was used for data analysis. Frequencies and percentages were computed for categorical. Mean and the standard deviation was analysed for continuous variables. Chi-square test was applied to study the effect of explanatory variables on the response variables where a p-value ≤ 0.05 is considered as a significant difference.

### Ethical Approval

(Ref: App # 0516-2020-LNH-ERC, Dated: April 23, 2020).

## RESULTS

A total of 184 patients were included who visited the hospital to seek medical services for their gastrointestinal issues during the COVID-19 lockdown phase. The mean age of the population was 42.7 years (±16.13) with patients falling within a range of 30 to 88 years. Out of 184 patients, n=94 (51.1%) were males. All patients had gastrointestinal issues with different comorbid conditions (Table-I)

**Table-I:** Demographic and Clinical Characteristics of Patients suffering from Cirrhosis during COVID-19 Pandemic.

Characteristics	Mean	SD

Age	42.7 years	±16.13

	Frequency n=184	(%)
***Age groups***		
10-35	70	(38%)
36-61	82	(44.6%)
62-88	32	(17.4%)
***Gender***		
Male	94	(51.1%)
Female	90	(48.9%)
***Co-morbidities***		
Diabetes Mellitus	01	(0.5%)
Hypertension	05	(2.7%)
Ischemic heart disease	01	(0.5%)
Other Issues (Including arthritis, anxiety, depression, thyroid, allergies etc.)	126	(68.5%)
Liver diseases	43	(23.4%)
Asthma	02	(1.1%)
***Active Complains***		
Abdominal pain	79	(42.9%)
Hepatic encephalopathy	04	(2.2%)
Fever	02	(1.1%)
Vomiting	08	(4.3%)
Diarrhea or Constipation	03	(1.6%)
Multiple Complains	53	(28.8)
Routine follow up	33	(17.9%)
No active complains	02	(1.1%)
***Reason for hospital visit***		
Active complain	147	(79.9%)
Follow-up visit	37	(20.1%)
***Length of complains***		
< 07 days	01	(0.5%)
08 – 28 days	138	(75%)
29 – 48 days	08	(4.3%)

The most common complaint was abdominal pain in n=79 (42.9%) followed by multiple gastrointestinal complaints, n=53 (28.8%). Another n=33 (17.9%) visited the hospital for their routine follow visit. (Table-I).

One forty-seven n=147 (79.9%) presented with active complaints with duration of complaint ranging from 8 days to 28 days. As shown in Table-II, n=79 (42.9%) of patients consulted a local general physician in their vicinity before coming to the hospital for their checkup, n=82 (44.6%) patients were attended by a local physician, n=10 (5.4%) patients visited the local GP for their issue resolution while n=25 (13.5%) patients visited ER for further management.

**Table-II:** Experiences of Patients related to Health Service Utilization during COVID-19 Pandemic.

Patient Experiences	Frequency (%) n=184
Visit to local GP before going to the hospital	79 (42.9%)
Availability of local physician	82 (44.6%)
Visit to local GP for issue resolution	10 (5.40%)
Visited ER for the complain	25 (13.5%)
Faced waiting in the ER	09 (4.90%)
Emergency treatment provided	20 (10.9%)
Unable to get ER admission because of limited space.	11 (6.00%)
***Number of attendants with the patients***
01	44 (23.9%)
02	113 (61.4%)
03-04	27 (14.6%)
Patients wearing masks	155 (84.3%)
***Type of mask used by the patients***
Surgical mask/N95	62 (33.7%)
Fabric mask	93 (50.5%)
Attendants wearing masks	151 (82.1%)
Avoided hospital due to fear of COVID-19	33 (17.9%)
***Mode of transportation***
Public	64 (34.8%)
Private	120 (65.2%)
Difficulty faced in finding public transport	83 (45.1%)
Satisfaction with the hospital service	171 (93.0%)

Out of 184 patients, n=170 (92.4%) had an attendant with them during the treatment at the hospital. Also shown in Table-II, n=155 (84.3%) patients wore face masks inside the hospital. Out of 184 patients, n=33 (17.9%) patients reported of having fear of visiting hospital due to COVID-19 pandemic. In response to the accessibility to the hospital, n=120 (65.2%) patients reported of reaching the hospital using private mode of transportation during COVID-19 pandemic. Of these 64 patients, n=83 (45.1%) patients faced difficulty in finding public transport to reach the hospital. In regards to satisfaction with the services rendered by the hospital during the COVID-19 pandemic n=171 (93.0%) patients expressed satisfaction with the services provided by the hospital during the lockdown phase.

The relation of demographic and clinical characteristics of patients with their experiences of health service utilization during the COVID-19 pandemic are described in Table-III. A statistically significant difference *p* < 0.001 was noted between the history of comorbidities and patient avoidance of seeking healthcare due to fear of COVID-19. Patients without comorbid conditions (51.5%) were more likely to avoid going to the healthcare facility due to fear of COVID-19 as opposed to patients with comorbidities (48.5%). Similarly, difficulties in finding public transportation were faced by those who had comorbidities (*p*=0.01).

**Table-III:** Association of Demographic and Clinical Characteristics with Patient Experiences.

	Avoided hospital due to the fear of COVID-19	P value	Difficulty faced in finding transport	P value
***Age***				
10-35	12 (36.4%)	0.575	34 (41.0%)	0.391
36-61	17 (51.5%)		38 (45.8%)	
62-88	04 (12.1%)		11 (13.2%)	
***Gender***				
Male	14 (42.4%)	0.272	37 (44.6%)	0.109
Female	19 (57.6%)		46 (55.4%)	
***Comorbidities***				
No	17 (51.5%)	<0.001	22 (26.5%)	0.01
Yes	16 (48.5%)		61 (73.5%)	
***Reason for visit***				
Active complain	26 (78.8%)	0.861	65 (78.3%)	0.628
Routine follow-up	07 (21.2)		18 (21.7%)	

## DISCUSSION

The current study explored the challenges faced by patients during pandemic and lockdown phase in getting health service utilization within a developing country with limited resources. A large body of evidence highlighted the role of the COVID-19 pandemic in disrupting primary, secondary as well as tertiary healthcare services across the globe^14^ with substantial negative implications on the health systems. ranging from collapsing of the systems to ethical issues in making decisions related to priority treatment due to extensive burden on the healthcare systems.^22^ Anticipating the scale of health services interference due to the COVID-19 pandemic, the World Health Organization (WHO) devised and circulated operational guidelines for the uninterrupted functioning of essential healthcare services during an infectious disease outbreak in March 2020, following the declaration of COVID-19 as a pandemic.^23^ In line with the developing situations and emerging evidence from academia and research institutions, the technical document was subsequently revised in the second quarter of 2021.^24^

Pakistan being a lower-middle-income country (LMIC) also faced significant challenges in the management of the COVID-19 pandemic and its massive burden on the health system was equally experienced by the population.^20^ The current study revealed that patients with chronic conditions were largely affected by the pandemic-related policies as routine follow-up visits to the clinicians and the healthcare facility are inherent to the management regime of such patients, whereas the accessibility to the services was reduced due to pandemic-related policies as well as the fear of the infection. This is evident from the current findings where approximately half (42%) of the patients visited a general healthcare practitioner in their area of residence instead of seeking care at the tertiary healthcare facility. These findings are incoherent with the results of a survey conducted in the United States where more than 50 percent of the patients admitted to postponing routine health care during the COVID-19 pandemic.^25^

In the present study, about one-third of patients with either active complaints or scheduled routine follow-up visits refrained from visiting the hospital due to fear of contracting COVID-19 infection. Fear of catching the infectious disease has been a major limiting factor in reducing health services utilization globally as well as in other endemics like Ebola virus and H1N1 outbreaks.^26^ In order to avoid delay in patient management and morbidity, it is recommended that telemedicine services should be widely accessible and integrated into the primary, secondary, and tertiary care during the pandemics to ensure effective continuity of care and other emergencies.

Interestingly, a significant proportion of the patients in the present study were accompanied by more than one attendant despite having institutional policies. In Pakistan most of the people lives in joint family system and also in a study by Roseland et al highlighted the role of families and support systems in the effective management of chronic diseases and improving patient care.^27^

Aside from the fear of disease, accessibility to health care services during health emergencies is another barrier underlying diminished health service utilization. Patients seeking health care often face hindrances due to the unavailability of public transportation augmented with restrictive social distancing policies. In our study, we noted that almost half of the patients (50%) encountered challenges in terms of access to healthcare facilities due to the unavailability of public transport. This shortage and absolute unavailability of public transportation during the initial phase of the pandemic manifested as inadequacies in the effective follow-up of the patients with chronic illness, despite these challenges 93% of the patients in our study were satisfied with the services provided by the healthcare facility.

### Limitations

As this study was conducted during the peak of the first COVID-19 wave in Pakistan which led to some challenges in recruiting patients for the study in addition to ensuring their safety. Due to these reasons, an exploratory research design was utilized. Moreover, findings from one healthcare facility may not be representative of the entire population therefore, cannot be generalized.

## CONCLUSION

The current study highlighted some challenges encountered by the patients suffering from gastrointestinal diseases. The accessibility to the healthcare services was notably affected due to pandemic-related policies and lockdown. Fear of contracting the COVID-19 diseases, issues in finding public transportation were found to be the major limiting factor causing rifts in health service utilization by the study population. Widely available telemedicine service might overcome these shortcomings and ensure continuity of quality care

### Author’s Contribution:

**HAK:** Data collection, data analysis. Writing first draft of manuscript.

**LK:** Conceptualization, protocol writing, and critical revision and final editing of manuscript
